# Supplementary material for: End Sequence Analysis Toolkit (ESAT) expands the extractable information from single-cell RNA-seq data
Source: Genome Res. 2016 Oct;26(10):1397–410. doi: 10.1101/gr.207902.116 (PMC5052061; doi:10.1101/gr.207902.116)
Supplement: Supplemental Material [file supp_gr.207902.116_Supp_Materials.tar.gz › ESAT-esat_v0.1/doc/html/classbroad_1_1pda_1_1seq_1_1segmentation_1_1_genome_with_gaps.html]

ESAT: broad.pda.seq.segmentation.GenomeWithGaps Class Reference


ESAT


- Main Page
- Related Pages
- Packages
- Classes
- Files

- Class List
- Class Index
- Class Hierarchy
- Class Members

All Classes Namespaces Files Functions Variables Enumerator Pages

- broad
- pda
- seq
- segmentation
- GenomeWithGaps

Public Member Functions |
List of all members

broad.pda.seq.segmentation.GenomeWithGaps Class Reference

Collaboration diagram for broad.pda.seq.segmentation.GenomeWithGaps:

[legend]

|  |  |
| --- | --- |
| Public Member Functions | |
|  | GenomeWithGaps (Collection< Alignments > gaps, String chr, int genomeSize, int windowSize, int startPosition) |
|  | |
| Collection< Annotation > | getGaps () |
|  | |
| IntervalTree< Annotation > | getCDNAs () |
|  | |
| boolean | hasNext () |
|  | |
| Gene | getNextWindow () |
|  | |
| Gene | getNextWindow (int position) |
|  | |

## Constructor & Destructor Documentation

|  |  |  |  |
| --- | --- | --- | --- |
| broad.pda.seq.segmentation.GenomeWithGaps.GenomeWithGaps | ( | Collection< Alignments > | *gaps*, |
|  |  | String | *chr*, |
|  |  | int | *genomeSize*, |
|  |  | int | *windowSize*, |
|  |  | int | *startPosition* |
|  | ) |  |  |

## Member Function Documentation

|  |  |  |  |  |
| --- | --- | --- | --- | --- |
| IntervalTree<Annotation> broad.pda.seq.segmentation.GenomeWithGaps.getCDNAs | ( |  | ) |  |

|  |  |  |  |  |
| --- | --- | --- | --- | --- |
| Collection<Annotation> broad.pda.seq.segmentation.GenomeWithGaps.getGaps | ( |  | ) |  |

|  |  |  |  |  |
| --- | --- | --- | --- | --- |
| Gene broad.pda.seq.segmentation.GenomeWithGaps.getNextWindow | ( |  | ) |  |

|  |  |  |  |  |  |
| --- | --- | --- | --- | --- | --- |
| Gene broad.pda.seq.segmentation.GenomeWithGaps.getNextWindow | ( | int | *position* | ) |  |

|  |  |  |  |  |
| --- | --- | --- | --- | --- |
| boolean broad.pda.seq.segmentation.GenomeWithGaps.hasNext | ( |  | ) |  |

---

The documentation for this class was generated from the following file:

- java/broad/pda/seq/segmentation/GenomeWithGaps.java


---

Generated on Fri Aug 15 2014 15:11:05 for ESAT by  

 1.8.7
